# Global and regional genetic association analysis of ulcerative colitis and type 2 diabetes mellitus and causal validation analysis of two-sample two-way Mendelian randomization

**DOI:** 10.3389/fimmu.2024.1375915

**Published:** 2024-11-22

**Authors:** Yan-zhi Hu, Zhe Chen, Ming-han Zhou, Zhen-yu Zhao, Xiao-yan Wang, Jun Huang, Xin-tian Li, Juan-ni Zeng

**Affiliations:** ^1^ The Second Affiliated Hospital of Hunan University of Chinese Medicine, Changsha, China; ^2^ Department of Thoracic Surgery, The Second Xiangya Hospital, Central South University, Changsha, China; ^3^ College of Traditional Chinese Medicine, Hunan University of Chinese Medicine, Changsha, China; ^4^ Laboratory of Vascular Biology and Translational Medicine, Medical School, Hunan University of Chinese Medicine, Changsha, China

**Keywords:** UC, T2DM, Mendelian randomization, LDSC analysis, LAVA analysis

## Abstract

**Background:**

Clinical co-occurrence of UC (Ulcerative Colitis) and T2DM (Type 2 Diabetes Mellitus) is observed. The aim of this study is to investigate the potential causal relationship between Ulcerative Colitis (UC) and Type 2 Diabetes Mellitus (T2DM) using LDSC and LAVA analysis, followed by genetic verification through TSMR, providing insights for clinical prevention and treatment.

**Methods:**

Genetic loci closely related to T2DM were extracted as instrumental variables from the GWAS database, with UC as the outcome variable, involving European populations. The UC data included 27,432 samples and 8,050,003 SNPs, while the T2DM data comprised 406,831 samples and 11,914,699 SNPs. LDSC and LAVA were used for quantifying genetic correlation at both global (genome-wide) and local (genomic regions) levels. MR analysis was conducted using IVW, MR-Egger regression, Weighted median, and Weighted mode, assessing the causal relationship between UC and diabetes with OR values and 95% CI. Heterogeneity and pleiotropy were tested using Egger-intercept, MR-PRESSO, and sensitivity analysis through the “leave-one-out” method and Cochran Q test. Subsequently, a reverse MR operation was conducted using UC as the exposure data and T2DM as the outcome data for validation.

**Results:**

Univariable and bivariable LDSC calculated the genetic correlation and potential sample overlap between T2DM and UC, resulting in rg = -0.0518, se = 0.0562, *P* = 0.3569 with no significant genetic association found for paired traits. LAVA analysis identified 9 regions with local genetic correlation, with 6negative and 3 positive associations, indicating a negative correlation between T2DM and UC. MR analysis, with T2DM as the exposure and UC as the outcome, involved 34 SNPs as instrumental variables. The OR values and 95% CI from IVW, MR-Egger, Weighted median, and Weighted mode were 0.917 (0.848~0.992), 0.949 (0.800~1.125), 0.881 (0.779~0.996), 0.834(0.723~0.962) respectively, with IVW *P*-value < 0.05, suggesting a negative causal relationship between T2DM and UC. MR-Egger regression showed an intercept of -0.004 with a standard error of 0.009, *P* = 0.666, and MR-PRESSO Global Test *P*-value > 0.05, indicating no pleiotropy and no outliers detected. Heterogeneity tests showed no heterogeneity, and the “leave-one-out” sensitivity analysis results were stable. With UC as the exposure and T2DM as the outcome, 32 SNPs were detected, but no clear causal association was found.

**Conclusion:**

There is a causal relationship between T2DM and UC, where T2DM reduces the risk of UC, while no significant causal relationship was observed from UC to T2DM.

## 1 Introduction

At present, the prevalence of chronic diseases such as diabetes, obesity, cardiovascular diseases, and inflammatory bowel disease poses a significant threat to global health. Diabetes Mellitus (DM) is a complex chronic illness characterized by glucose metabolic dysfunction caused by either absolute or relative insulin deficiency. The global incidence of diabetes is on the rise, with a total prevalence of 10.5% among adults aged 20-79 years, reaching 537 million diabetic patients worldwide by 2021, and it’s estimated to increase to 783.2 million by 2045 (1). Type 2 Diabetes Mellitus (T2DM) is the most common form, accounting for approximately 90-95% of all cases. T2DM typically presents with various comorbidities and long-term complications, including cardiovascular diseases, retinopathy, nephropathy, and neurological disorders, which have garnered significant attention. Moreover, the emergence of new complications such as COVID-19, pulmonary fibrosis, and gastrointestinal diseases is increasingly common (2). The onset of T2DM is closely associated with genetic factors, aging, and unhealthy lifestyle habits. It’s generally believed that the pathophysiology of T2DM is rooted in impaired insulin responsiveness, known as insulin resistance (IR) (3), coupled with inadequate insulin secretion. Research indicates that the development of insulin resistance is linked to endotoxemia, chronic inflammatory responses, short-chain fatty acid, and bile acid metabolism, with a notable imbalance in the gut microbiota of diabetic patients (4). This dysbiosis of gut microbiota, resulting from changes in microbial composition, bacterial metabolic activity, or local distribution, can trigger a decline in immune function, chronic inflammatory responses, and an imbalance in energy metabolism, leading to metabolic disorder and insulin resistance, ultimately contributing to the development of T2DM (5).

Ulcerative colitis (UC), a chronic, non-specific inflammatory bowel disease (6), has increasingly become a common and intractable condition in the digestive system. Its primary clinical manifestations include recurrent diarrhea with mucosal bloody stool, with or without abdominal pain. Inflammation and ulcers can appear in various sections of the large intestine, predominantly affecting the rectum and sigmoid colon, and occasionally the ileum, leading to backwash ileitis. This condition can cause anemia, liver disease, arthropathy, mucocutaneous diseases, and eye disorders. Severe cases may develop toxic megacolon, intestinal perforation, and cancer. UC, along with Crohn’s disease (CD), is categorized as inflammatory bowel disease (IBD), frequently observed in individuals with a high-fat diet preference (7). Ulcerative colitis (UC) has now emerged as a pervasive global health challenge, with its epidemiological trends evolving continuously. Research highlights a rapid escalation in the incidence of UC within low to middle-income nations. The disease manifests with comparable frequency in both males and females, predominantly affecting individuals aged between 2 and 40 years. However, there’s an increasing prevalence of UC in the population over 60 years of age, who account for 20% of newly diagnosed cases. These shifting patterns underscore the imperative need for refining and globalizing preventive and therapeutic strategies for UC, to effectively address its dynamic disease burden (8). Past studies have attributed the etiology of UC to genetic (9), environmental (10), dietary (11, 12), and psychological factors (13). The pathogenesis primarily involves genetic predisposition, gut microbiome imbalance, immune response irregularities, imbalance of pro-inflammatory and anti-inflammatory factors, aberrant signaling pathways, hypercoagulable blood state, intestinal epithelial cell apoptosis, necroptosis, long non-coding RNA, and proteomics. Theories such as “autophagy-cytokine-bacteria-UC” and “intestinal loop poisoning” have been proposed (14–23). Clinically, patients with coexisting UC and T2DM exhibit disease-related characteristics, higher hospitalization rates, increased risk of concurrent infections, and poorer prognosis (1). When T2DM coexists with UC, fluctuations in blood sugar levels, combined with intestinal lesions in patients, hinder the intake and absorption of nutrients and accelerate their loss. Particularly during active phases of UC, symptoms such as fever and diarrhea can increase the body’s metabolism, leading to an insufficient supply of nutrients. Consequently, patients may exhibit varying degrees of malnutrition, significantly impacting their physical health and quality of life.

Linkage Disequilibrium Score Regression (LDSC) is a statistical method used in genetic research (24), widely applied in Genome-Wide Association Studies (GWAS). Its primary purpose is to estimate the degree of genetic influence on specific traits or diseases, known as genetic correlation. LDSC’s key feature is the use of linkage disequilibrium (LD) scores to correct associations between multiple genetic markers. LD describes the co-inheritance patterns of genetic markers (like Single Nucleotide Polymorphisms, SNPs) within a population. In GWAS, the abundance of genetic markers and their LD can cause statistical confusion, affecting accurate estimations of genetic correlation. LDSC analysis enables researchers to more precisely estimate the contribution of genetic variations to specific traits or disease risks. This method is significant in understanding the genetic background of complex traits, revealing genetic risk factors, and providing a theoretical foundation for personalized medicine and gene therapy.

LAVA (Local Analysis of Variant Association) refers to a statistical method or tool used in genetics and bioinformatics (25). It is designed to analyze genetic variations, such as Single Nucleotide Polymorphisms (SNPs), whether in localized regions or across the entire genome. The primary aim of this analysis is to identify associations between genetic variations and specific traits or diseases. Employed in Genome-Wide Association Studies (GWAS), researchers use LAVA to pinpoint genetic variations linked to particular traits or diseases. Focusing on localized areas, LAVA provides in-depth insights, aiding researchers in accurately locating specific variants or groups of variants contributing to disease risks or manifestations. LAVA typically involves the use of statistical algorithms and computational tools to process large genomic datasets and can be integrated with other bioinformatics methods to enhance the analysis and interpretation of genetic data.

**Figure 1:**
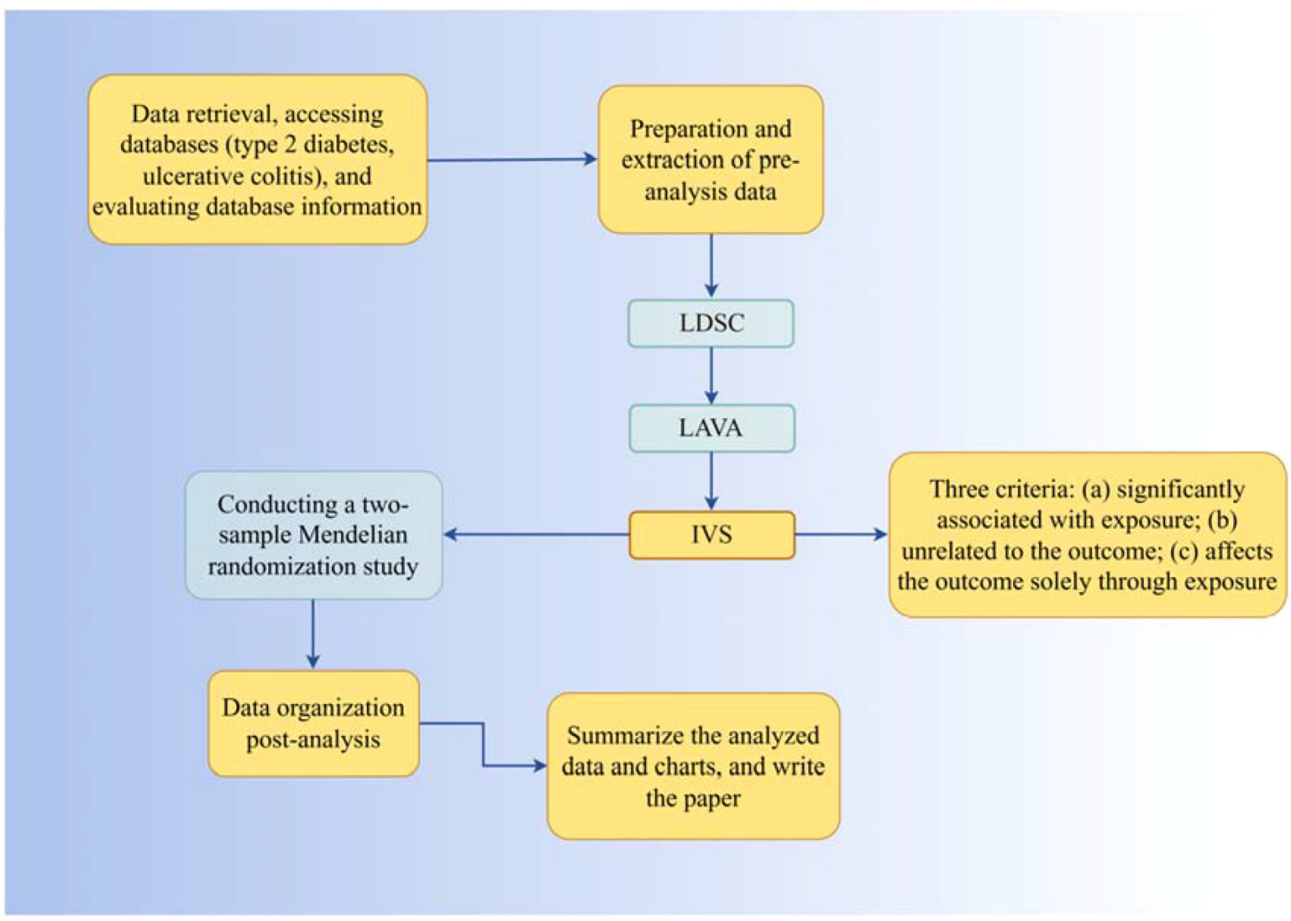
The process flowchart of the analysis.

Randomized Controlled Trials (RCTs), due to various constraints, are challenging to implement effectively in clinical settings. Observational experimental methods, influenced by confounding factors and reverse causality, tend to yield biased results with relatively low credibility. In 1986, Martijn B. Katan proposed that different alleles determine varying Apolipoprotein E subtypes, influencing cancer incidence rates through cholesterol level regulation, laying the groundwork for the concept of Mendelian Randomization (MR) (26). In 2004, Thomas and Conti introduced the use of genetic information as instrumental variables for causal inference in epidemiology (27). MR employs Single Nucleotide Polymorphisms (SNPs), or genetic variants, as instrumental variables. Based on Mendel’s laws of inheritance, genetic variations are randomly distributed to offspring during meiosis and remain unchanged thereafter. This directional and invariant nature of MR reduces the influence of reverse causation and confounding factors, as compared to observational studies (28, 29). Two-sample Mendelian Randomization (TSMR), involving data from two independent databases, enhances sample size and the availability of exposure and outcome sources. Recently, with the release of numerous large-scale Genome-Wide Association Studies (GWAS), MR has become a viable method for assessing disease risk factors. To circumvent the limitations of observational studies, we use LDSC and LAVA analysis to explore the genetic correlation between T2DM and UC, followed by MR analysis for bidirectional causal verification, investigating potential mechanisms influencing this correlation. All original studies have received ethical approval, so additional ethical approval or informed consent for this research is not required. The process flowchart of the analysis is shown in **Figure 1**.

## 2 Materials and methods

### 2.1 Study design

This study employs LDSC (LD Score Regression) and LAVA (Local Analysis of Variant Association) to estimate the genetic correlation between Type 2 Diabetes Mellitus (T2DM) and Ulcerative Colitis (UC). Utilizing T2DM as the exposure factor, Single Nucleotide Polymorphisms (SNPs) significantly related to T2DM are used as instrumental variables (IVs), with UC as the outcome variable. The process involves reverse operation verification using the TwoSampleMR package in R for causal association analysis, including Cochran Q heterogeneity test, pleiotropy test, and sensitivity analysis to validate the results. The selection of IVs is based on three criteria: significant association with T2DM, irrelevance to UC, and exclusive influence on UC through T2DM. These criteria are independent and indispensable, determining the suitability of IVs for analysis.

### 2.2 Source of data

All data in this study are sourced from publicly available Genome-Wide Association Studies (GWAS) and the IEU GWAS database. We retrieved GWAS summary statistics, selecting SNPs significantly associated with T2DM as genetic instrumental variables from the IEU GWAS database. For Ulcerative Colitis (UC), GWAS summary statistics related to UC were selected from large-scale published GWAS meta-analyses, extracting gene outcome associations (30). The UC data includes a sample size of 27,432 individuals with 8,050,003 SNPs, while the T2DM data comprises 406,831 individuals with 11,914,699 SNPs.

### 2.3 LDSC analysis

To assess the shared genetic components between Type 2 Diabetes Mellitus (T2DM) and Ulcerative Colitis (UC), we conducted a global genetic correlation analysis using bivariate linkage disequilibrium (LD) score regression (LDSC), with values ranging from -1 to 1. LDSC estimates the heritability of individual traits or genetic correlation between traits by constructing a regression relationship between LD scores and GWAS test statistics. LD scores are calculated using European ancestry reference data from the 1000 Genomes Project, limited to 1.2 million well-qualified HapMap3 SNPs, excluding SNPs in the MHC region due to their complex LD patterns affecting genetic correlation estimates. To address unknown sample overlaps in LDSC analysis, we did not restrict the intercept term, using it to assess potential population stratification in individual trait GWAS or sample overlap between pairs of GWAS data. A significant threshold is determined as a *P*-value less than 0.05 after false discovery rate (FDR) correction (Benjamini-Hochberg method), equating to a q value < 0.05.

### 2.4 LAVA analysis

The global genetic correlation estimated by LDSC originates from the aggregate information of all variations in the genome. However, due to the complexity of genetic variations and their associations with diseases, different regions contribute variably in magnitude and direction to the genetic correlation. Moreover, significant disparities exist in the genetic correlation of two traits in different regions, particularly where opposing regional genetic correlations may neutralize each other. This can reduce the global genetic correlation between traits, obscuring potential pleiotropic effects. Therefore, we employ Local Analysis of Variant Association (LAVA) to estimate the genetic correlation between T2DM and UC in independent local regions of the genome (25). LAVA is conducted within 2,495 independent LD blocks previously delineated, with LD estimations based on the 1000G EUR reference. The significant threshold is set as a P-value less than 0.05 following false discovery rate (FDR) correction (Benjamini-Hochberg method), corresponding to a q value < 0.05.

Based on the chromosomal segments identified by LAVA analysis (details are provided in **Supplementary Material**), we conducted Bayesian colocalization analysis on diseases showing significant local genetic correlation after FDR multiple correction, in order to further clarify whether the two phenotypes share the same causal variant within a given region. Unfortunately, within the CHR segments provided by the LAVA analysis, no significant shared causal variant loci were observed between T2DM and UC (see Appendix for details), suggesting that although there may be some common genetic factors between T2DM and UC, they may be caused by different genetic variations on the studied chromosomal segments. That is, the two traits may be controlled by different regulatory regions of the same gene, appearing to be genetically related, but showing no significant shared genetic variation at the expression level, hence no colocalization signal was detected in this segment. Combined with the positive results of the MR analysis, it can be explained that the relationship between the studied traits is entirely due to the impact of exposure on the outcome. Of course, considering the robustness of the LAVA analysis results, future studies will focus on further explaining these results by increasing sample size, integrating other biological data, using more precise statistical methods, and possible experimental validation.

### 2.5 Selection and validation of IVs

For Type 2 Diabetes Mellitus (T2DM) exposure, the selection of Instrumental Variables (IVs) starts with T2DM’s database. The steps to determine the included IVs are as follows: 1. Initially select SNPs that meet the significance threshold (*P* < 5×10^-8); 2. Exclude SNPs in linkage disequilibrium (LD), mainly based on the distance and r^2 value between each SNP (r^2 < 0.01, distance > 10000kb); 3. Further remove palindromic SNPs from the determined SNP list, especially those with lower effect allele frequency (< 0.58) in the outcome, as it’s challenging to discern the strand orientation of such SNPs. 4. Eliminate the influence of other confounding factors. Considering UC’s complexity, it’s crucial to account for common confounders like Irritable Bowel Syndrome, hyperlipidemia, body weight, and fatty liver, which might affect its occurrence as intermediate phenotypes. To avoid IVs affecting the outcome through common confounders, verify the SNP through the Pheno Scanner database (version 2: http://www.phenoscanner.medschl.cam.ac.uk/), delete SNP: rs11651052 (Prostate cancer), rs2844623 (Crohn’s disease), and similarly, for the reverse scenario.

### 2.6 MR analytics

This study’s statistical analysis is based on R software (version 4.3.0, R Foundation for Statistical Computing, Vienna, Austria). The focal analysis relies on the Two Sample MR (TSMR) R package developed by Gibran Hemani and colleagues (31, 32). We employed four methods to estimate effects: Inverse Variance Weighted method (IVW) (33), MR-Egger regression (34), Weighted median (35), and Weighted mode (36). The primary outcome measure is the Odds Ratio (OR), including a 95% Confidence Interval (CI). Statistical results encompass the overall effect size, standard error (yielding the final OR and 95% CI), and significance values, with a default two-sided test *P*<0.05 considered statistically significant. Scatter plots derived from statistical tables illustrate these results. MR-PRESSO and MR-Egger regression methods calculate the magnitude of pleiotropy, presented graphically via weighted linear regression, where the intercept’s absolute value indicates the extent of pleiotropy; a pleiotropy *P*>0.05 is not statistically significant (37). Sensitivity analysis employs the “leave-one-out” approach from the R package, reanalyzing results after sequentially excluding individual SNPs and visualizing the impact of each SNP on outcomes via forest plots to assess result stability. Heterogeneity is tested using Cochran’s Q test, with a *P*>0.05 indicating no significant heterogeneity, and results are presented in statistical tables.

## 3 Result

### 3.1 Genetic correlation analysis

We employed both LDSC and LAVA to quantify the pairwise genetic correlation at global (i.e., across the entire genome) and local (i.e., within specific genomic regions) levels. The LAVA analysis estimated local genetic correlations across 2,495 genomic regions.

**Figure 2:**
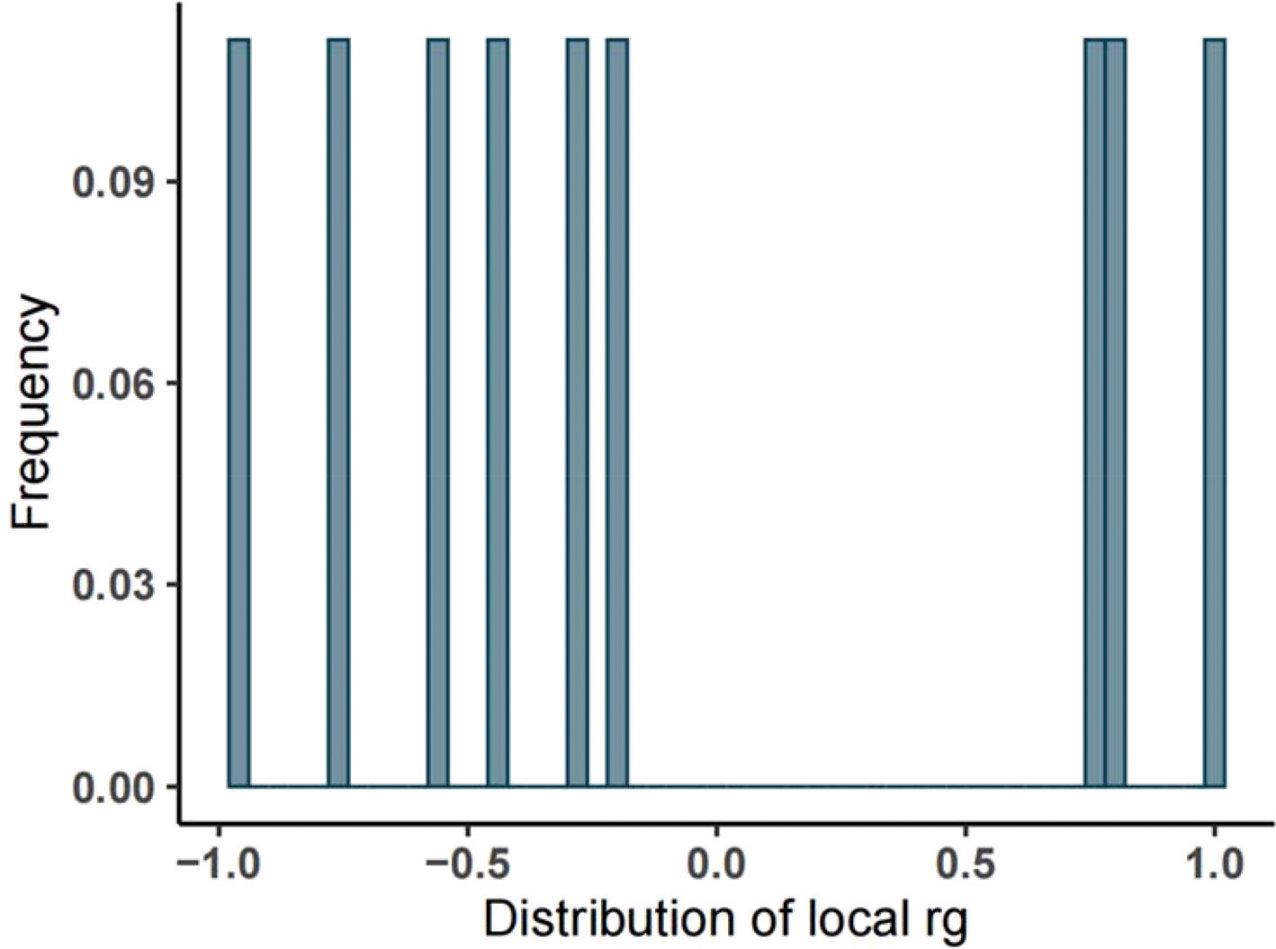
Frequency distribution of local heritability of T2DM and UC. (Note: The abscissa represents the heritability, the ordinate represents the frequency, and the rg ranges from -1 to 1).

Initially, we utilized bivariate LDSC to calculate the genetic correlation and potential sample overlap between T2DM and UC. After adjusting all *P*-values (FDR *P*<0.05), no significant genetic associations were found for the paired traits, with rg = -0.0518, se = 0.0562, *P* = 0.35699, as shown in **Table 1**. Subsequently, our LAVA analysis of local genetic correlations indicated, after FDR multiple adjustments, that 9 regions exhibited local genetic correlations for at least one pair of traits (**Figure 2**), with 33.33% positive and 66.67% negative correlations. Specifically, 6 regions were negatively and 3 positively significantly associated, leading us to conclude that LAVA results show a negative correlation between T2DM and UC incidence. The inheritance of disease is a multifaceted and intricate process, interwoven with a multitude of genetic and environmental interactions. Global analysis through LDSC, constrained by sample sizes, statistical methodologies, or the inherent genetic complexity of the diseases themselves, sometimes fails to significantly reveal correlations. In contrast, LAVA’s local analysis, with its focus on specific genes or regions, possesses the capability to unearth more profound genetic mechanisms. Certain genetic effects may only be significant in specific gene areas or populations, nuances that global analysis might overlook, while local analysis can investigate these effects with greater precision. Moreover, global analysis might be influenced by sample bias, such as insufficient diversity or selective recruitment, potentially obscuring the true genetic associations. On the other hand, local analysis often employs more representative samples and more precise genetic markers. These factors could account for the negative findings in LDSC analysis versus the negative correlation in LAVA results.

**Table 1:** LDSC results.

Genetic correlation				
Trait pair	Genetic correlation (SE)	P value for LDSC	Intercept (SE)	P value for Intercept
T2DM-UC	-0.0518 (0.0562)	0.3569	0.0112 (0.0067)	0.095

### 3.2 Bidirectional MR analysis of UC by T2DM

#### 3.2.1 Status of instrumental variables

Initially, with Type 2 Diabetes Mellitus (T2DM) as the exposure and Ulcerative Colitis (UC) as the outcome, we utilized R software to select genome-wide significant SNP loci according to our screening criteria. To mitigate the impact of common confounders via Instrumental Variables (IVs), we further validated these SNPs through the Pheno Scanner database, resulting in 34 SNPs as IVs. Similarly, with UC as the exposure and T2DM as the outcome, we identified 32 SNPs as IVs.

#### 3.2.2 MR analysis results

The Mendelian Randomization (MR) analysis for the relationship between Type 2 Diabetes Mellitus (T2DM) and Ulcerative Colitis (UC) was conducted using the TwoSampleMR package, employing methods such as Inverse Variance Weighted (IVW), MR-Egger regression, Weighted median, and Weighted mode. The results, detailed in **Table 2**, reveal Odds Ratios (OR) and 95% Confidence Intervals (CI) as follows: 0.917 (0.848~0.992), 0.949 (0.800~1.125), 0.881 (0.779~0.996), and 0.834(0.723~0.962). These findings indicate that having T2DM reduces the risk of developing UC, with *P*-values from the four tests being 0.031, 0.550, 0.042, and 0.018, respectively. The IVW result, significant at *P*<0.05, and the consistent direction of β values across all methods, validate the conclusion that T2DM lowers the risk of UC, suggesting a causal relationship. The MR-PRESSO result, with a *P*-value < 0.05, reinforces the robustness of this positive outcome, further substantiating the negative correlation found in the LAVA analysis.

**Table 2:** Results of MR Analysis of T2DM versus UC.

Method	BETA	SE	OR (95% CI)	P value
IVW	-0.086	0.040	0.917 (0.848~0.992)	0.031
MR-Egger	-0.053	0.087	0.949 (0.800~1.125)	0.550
Weighted median	-0.127	0.063	0.881 (0.779~0.996)	0.042
Weighted mode	-0.181	0.073	0.834(0.723~0.962)	0.018

The Mendelian Randomization (MR) analysis of Ulcerative Colitis (UC) on Type 2 Diabetes Mellitus (T2DM) was performed using the same methodology, with results presented in **Table 3**. The Odds Ratios (OR) and 95% Confidence Intervals (CI) are reported as 1.018 (0.991~1.046), 0.993 (0.911~1.083), 1.030 (0.995~1.065), and 1.039 (0.985~1.096). The P-values for the four tests are 0.187, 0.879, 0.096, and 0.171, respectively. With the IVW result being greater than 0.05, the difference is not statistically significant. Hence, the MR analysis suggests no evident causal relationship between the occurrence of UC and the development of T2DM.

**Table 3:** Results of MR Analysis of UC for T2DM.

Method	BETA	SE	OR (95% CI)	P value
IVW	0.018	0.014	1.018 (0.991~1.046)	0.187
MR-Egger	-0.065	0.044	0.993 (0.911~1.083)	0.879
Weighted median	-0.005	0.017	1.030 (0.995~1.065)	0.096
Weighted mode	-0.002	0.027	1.039 (0.985~1.096)	0.171

#### 3.2.3 Sensitivity analysis result

**Figure 3:**
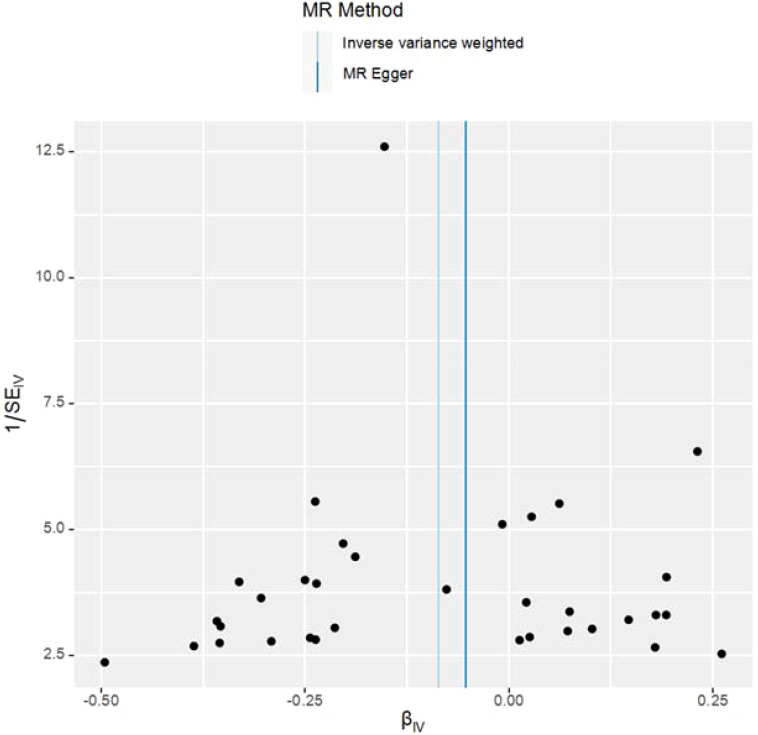
Funnel plot of the results of heterogeneity test for MR Method analysis.

This study meticulously adhered to the selection criteria for instrumental variables, thus reducing the likelihood of false-negative results. For the MR analysis of T2DM’s impact on UC, heterogeneity tests were conducted. The Q-values and Q*P*-values for IVW and MR-Egger were 19.933 (0.952) and 20.122 (0.962), respectively, both exceeding 0.05, indicating no significant heterogeneity. The results have been visualized in **Figure 3**.

**Figure 4:**
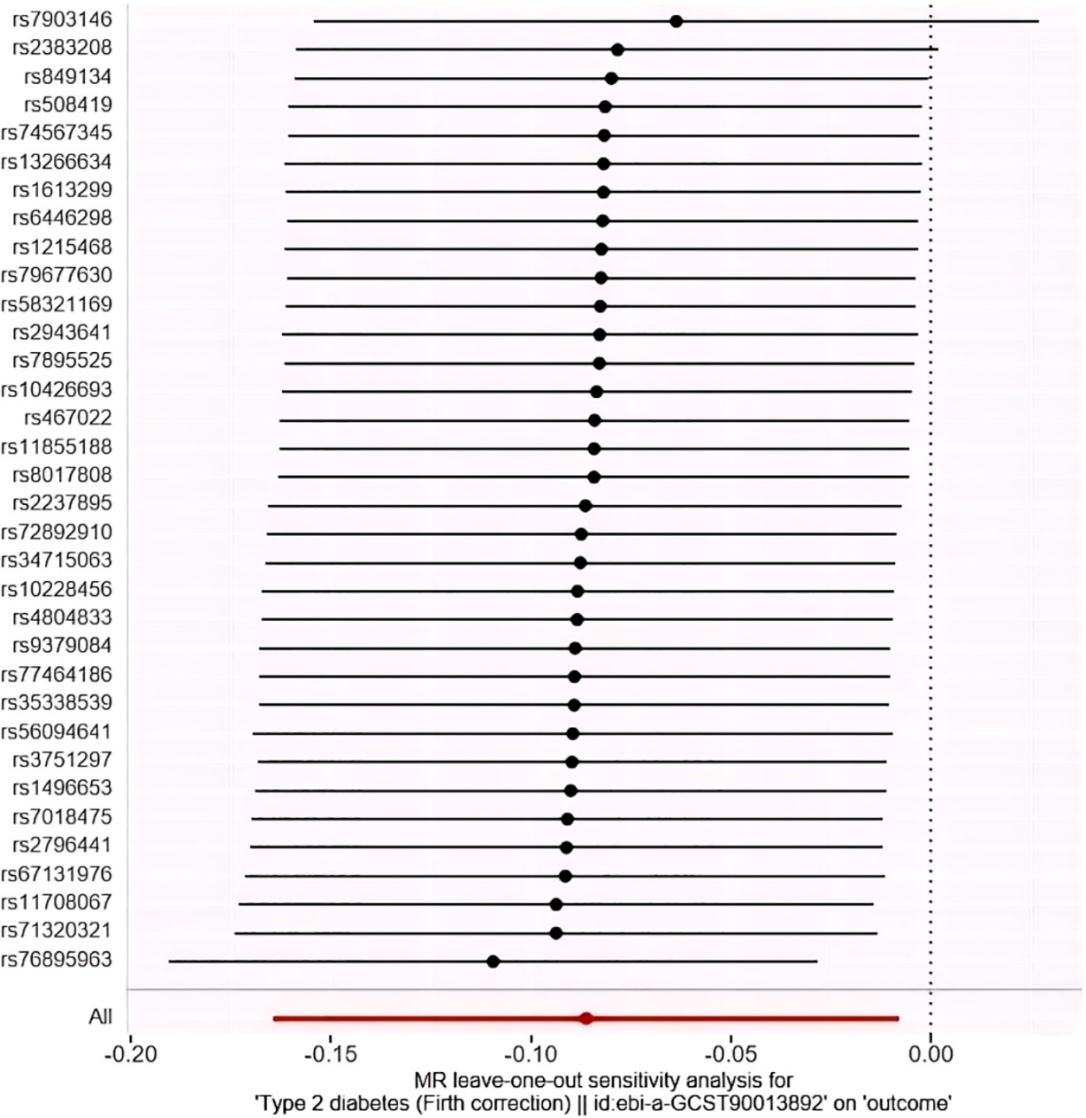
“Leave-one-out” method to visualize the results of IVW method. (Leave-one-out analysis refers to the MR Analysis after eliminating SNPS one by one, generally to see whether they are all significant, or whether the mean value is greater than/less than 0/1).

**Figure 5:**
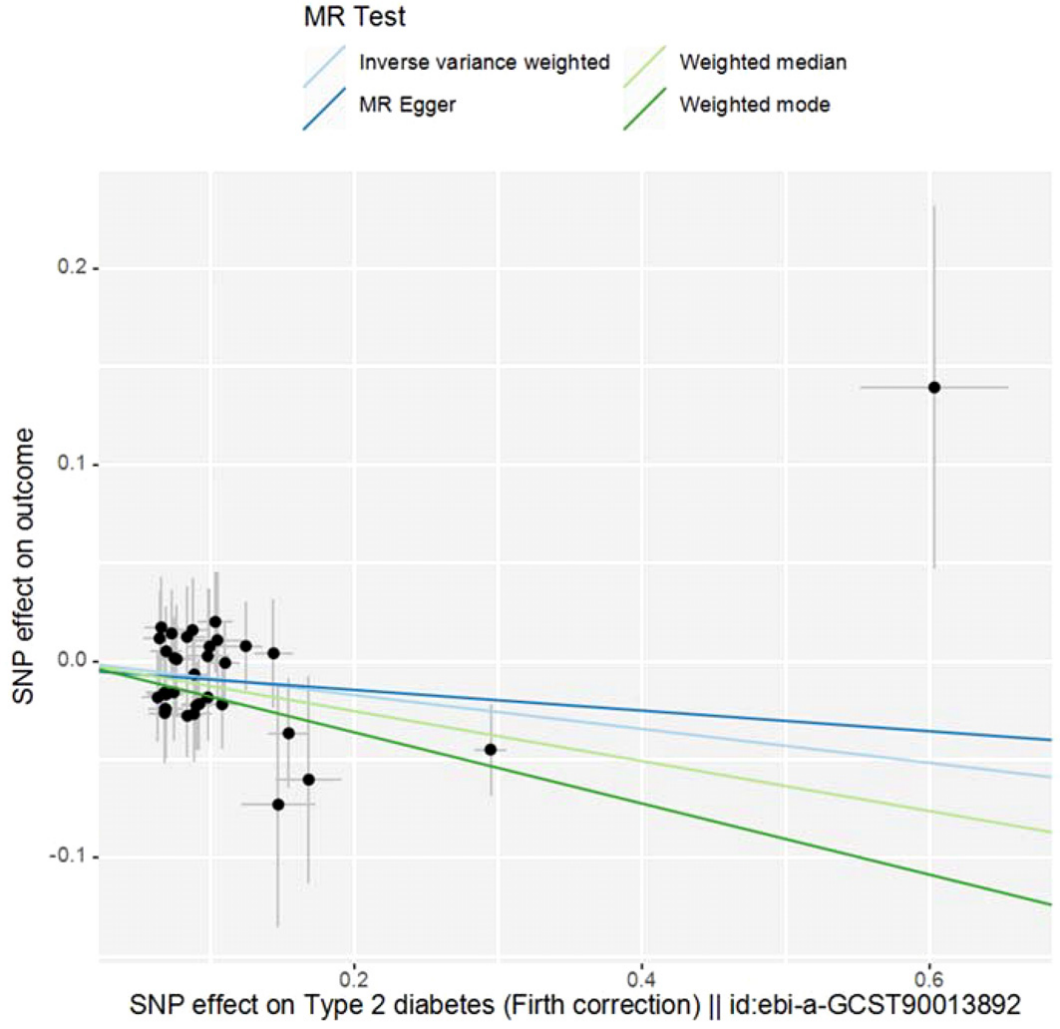
Scatter plot of MR Analysis. (Note: The X-axis represents the SNP effect on exposure, and the Y-axis represents the SNP effect on outcome. Slope less than 0, indicating that the exposure is a favorable factor for the outcome).

The study employed MR-Egger regression’s intercept to assess potential pleiotropy. The Egger-intercept value was -0.004, close to zero, with SE = 0.009 and *P* = 0.666, suggesting minimal pleiotropy. MR-PRESSO, supplementing the primary IVW results, showed a consistent direction of the Causal (beta effect value), with a *P*-value less than 0.05. The Global Test *P*value of 0.954 indicates no horizontal pleiotropy, affirming the MR results are free from multi-effect interference. Sensitivity analysis using the “Leave-one-out” method visualized the IVW results in **Figure 4**. After sequentially excluding individual SNPs, the remaining SNPs’ IVW effect values showed no significant fluctuations, aligning closely with the red dot in the figure, and all *P*-values were above 0.05. This indicates the absence of SNPs with strong influence in the instrumental variables, confirming the stability and reliability of the IVW results. No outliers were detected in the MR-PRESSO process. The final MR results are visualized in **Figure 5**.

Based on the chromosomal segments identified by LAVA analysis (details are provided in Appendix), we conducted Bayesian colocalization analysis on diseases showing significant local genetic correlation after FDR multiple correction, in order to further clarify whether the two phenotypes share the same causal variant within a given region. Unfortunately, within the CHR segments provided by the LAVA analysis, no significant shared causal variant loci were observed between T2DM and UC (details see **Supplementary Figures 1–9**), suggesting that although there may be some common genetic factors between T2DM and UC, they may be caused by different genetic variations on the studied chromosomal segments. That is, the two traits may be controlled by different regulatory regions of the same gene, appearing to be genetically related, but showing no significant shared genetic variation at the expression level, hence no colocalization signal was detected in this segment. Combined with the positive results of the MR analysis, it can be explained that the relationship between the studied traits is entirely due to the impact of exposure on the outcome. Of course, considering the robustness of the LAVA analysis results, future studies will focus on further explaining these results by increasing sample size, integrating other biological data, using more precise statistical methods, and possible experimental validation.

## 4 Discussion

Significant progress in T2DM and UC comorbidity research has emerged, leveraging genomics and metabolomics. This work elucidates genetic and epigenetic links, and enhances our understanding of their epidemiology, pathogenesis, and therapeutic strategies.

The link between Ulcerative Colitis (UC) and Type 2 Diabetes Mellitus (T2DM) risk is inconsistent across studies. Jess et al.’s Danish cohort study and Kang’s South Korean database analysis found an increased T2DM risk in UC patients (38–40), while a Taiwanese study did not (41). Surgical procedures, especially left-sided colon resections, may also heighten T2DM risk (42). These variations may stem from different study designs and populations. Our data hints at an inverse causal relationship from T2DM to UC, but not vice versa, as supported by LAVA and MR analyses. Both conditions share pathophysiological traits like gut microbiota disruption, epithelial barrier dysfunction, and inflammation (43, 44).

Our study, employing LDSC, LAVA, and MR methods, indicates a decreasing incidence of Ulcerative Colitis (UC) in patients with Type 2 Diabetes Mellitus (T2DM). Tseng CH’s research highlights a dose-response relationship between metformin use and a diminished risk of UC, which could account for this observed trend (45). Metformin’s potential to lower UC risk is thought to be mediated through its effects on improving insulin resistance, modulating gut microbiota, and reducing inflammation. It may alleviate intestinal inflammation in UC by suppressing pro-inflammatory cytokines and chemokines (46–49). Furthermore, metformin’s ability to increase the presence of Akkermansia muciniphila, a bacterium associated with UC remission (50–53), suggests its contributory role in the lower incidence of UC among T2DM patients.

The use of Glucagon-Like Peptide-1 (GLP-1) may also play a role in the observed decrease in UC incidence among T2DM patients. GLP-1, a mild insulinotropic hormone, has diverse pharmacological effects, including stimulating insulin release in response to glucose, slowing stomach emptying, and reducing appetite (54). Modified GLP-1 receptor agonists, with improved potency and longer action, are effective in treating T2DM (55). Wenrui Wang’s research indicates that GLP-1 can significantly reduce UC by inhibiting pro-inflammatory mediators, protecting against intestinal damage, and mitigating gut microbiota imbalance caused by DSS (56). Thus, GLP-1 treatment in T2DM patients might contribute to lower UC rates.

However, the impact of metformin and GLP-1 receptor agonists may involve gene-environment interactions. Genetic variants could affect UC risk in the context of drug exposure. Polygenic risk scores (PRS) might identify a shared genetic risk for T2DM and UC, with certain variants influencing medication responses. Since UC is immune-mediated and T2DM involves chronic inflammation, genetic factors could regulate immune responses, affecting both conditions. Metformin and GLP-1 receptor agonists may modify UC risk by altering immunoregulatory gene expression or function.

Irisin, a 112-amino acid peptide produced by skeletal muscle and derived from FNDC5, plays a pivotal role in a range of physiological responses and may mediate the connection between neurological health and physical activity (57). Huangfu Lixin’s (58) research on UC patients found significantly reduced FNDC5 and Irisin levels in colonic tissue and serum, respectively, with Irisin inversely correlating with IL-12 and IL-23, echoing earlier findings of its link to inflammation (59, 60).

The study also identified significant gut microbiota imbalances in active UC, characterized by decreased lactobacillus and increased enterococcus, which correlated with inflammation severity. Irisin levels negatively associated with enterococcus and positively with lactobacillus, suggesting its role in UC pathogenesis.

In T2DM patients, higher FNDC5 levels were linked to older age and poor glycemic control (61). This proposes that elevated FNDC5 and Irisin’s anti-inflammatory effects in T2DM may reduce cytokines, enhance gut microbiota health, and protect against UC by preventing dysbiosis and pathogen invasion.

Studies indicate that T2DM patients have significantly higher serum levels of TGF-β1 compared to non-diabetic individuals (62), Hefini conducted a study on the serum Transforming Growth Factor-β (TGF-β) levels in a cohort of 45 patients diagnosed with Type 2 Diabetes. The research revealed a substantial positive correlation between the onset of macroalbuminuria and the duration of diabetes. Furthermore, the analysis indicated that the serum TGF-β concentrations were substantially elevated in patients exhibiting macroalbuminuria (63). TGF-β, an anti-inflammatory cytokine primarily produced by activated T lymphocytes, B lymphocytes, and monocytes, promotes the synthesis and secretion of matrix proteins and epithelial repair (64), thereby potentially reducing the incidence of UC. Additionally, T2DM patients may adopt a healthier diet, opting for low-sugar and low-fat options, which could further decrease the potential risk of UC.

Addressing the needs of patients co-managing UC and T2DM presents a nuanced challenge in medical practice, owing to the intricacies in treatment and prognosis of these conditions. Stabilizing UC necessitates anti-inflammatory and immunomodulatory treatments, coupled with vigilant blood sugar level management. Moreover, prevention of cardiovascular diseases is critical, potentially entailing stricter control of blood pressure and lipid levels. Early identification of the interplay between UC and T2DM enables physicians to more accurately assess patient risk and tailor preventative and treatment strategies. Early interventions in high-risk groups, such as dietary improvements and increased physical activity, can significantly reduce disease risk. Comprehensive medical strategies, augmented by guidance on disease management, enhance patient adherence to treatment, thereby optimizing therapeutic outcomes.

## 5 Conclusion

In summary, this study utilized LDSC, LAVA, and TSMR analyses to explore the association between UC and T2DM. The results suggest a negative correlation, indicating that T2DM may reduce the risk of UC. This was further supported by genetic validation analysis. Factors contributing to this result include the use of metformin and GLP-1 in T2DM patients, increased Irisin secretion due to elevated serum FNDC5 levels, elevated serum TGF-β1, and dietary changes in T2DM patients. No significant causal association has been observed between UC and the risk of developing Type 2 Diabetes Mellitus. Based on publicly available GWAS data, this research avoids biases common in RCTs and observational studies, unlike previous observational studies. The findings are further supported by heterogeneity checks, with no evidence of heterogeneity or pleiotropy, and the “leave-one-out” sensitivity analysis confirms the reliability of the results. This research, unrestricted by ethical and financial constraints, provides insights into epidemiological etiology and may inform strategies for reducing the severity of UC in T2DM patients and aid in clinical treatment and risk prediction for patients with both conditions.

## 6 Deficiency and prospect

This study also has certain limitations, primarily including the following aspects: (1) The GWAS included in this study mainly comes from the European population, so the results may not necessarily match other ethnicities, which requires further GWAS of more diverse ethnicities to validate the results or discover new applicable loci. (2) The GWAS data extracted for this analysis does not have stratified analysis results for gender, age, duration, etc., so specific information cannot be studied. Based on a large sample research design, obtaining more instrumental variables can enhance the reliability of the results, and subsequent research can delve into more studies on Asian populations. In the future, specific causal mechanisms between T2DM and UC can be further explored through experimental methods, including cellular biology factors, physicochemical factors, genetic factors, immune factors, etc. Addressing the needs of patients co-afflicted with Ulcerative Colitis (UC) and Type 2 Diabetes Mellitus (T2DM) presents a significant challenge in medical practice, stemming from the complexity inherent in the treatment and prognosis of both conditions. To maintain stability in UC, patients require anti-inflammatory and immunomodulatory therapies, while simultaneously managing glycemic levels. Additionally, the prevention of cardiovascular diseases is indispensable, potentially including stricter blood pressure and lipid control. Early identification of the interplay between UC and T2DM aids physicians in more accurately assessing patient risk and devising tailored prevention and treatment strategies. For high-risk patient groups, early interventions, such as dietary improvements and increased physical activity, can effectively mitigate disease risk. Comprehensive medical measures, coupled with guidance on disease management for patients, can enhance treatment adherence, thereby optimizing therapeutic outcomes.

## Data Availability

The datasets presented in this study can be found in online repositories. The names of the repository/repositories and accession number(s) can be found in the article/**Supplementary Material**.
