# Supplementary material for: Global and regional genetic association analysis of ulcerative colitis and type 2 diabetes mellitus and causal validation analysis of two-sample two-way Mendelian randomization
Source: Front Immunol. 2024 Nov 22;15:1375915. doi: 10.3389/fimmu.2024.1375915 (PMC11621067; doi:10.3389/fimmu.2024.1375915)
Supplement: Supplementary file 3 [file DataSheet3.docx]

Supplementary Figures 1-9

Bayesian colocalization results


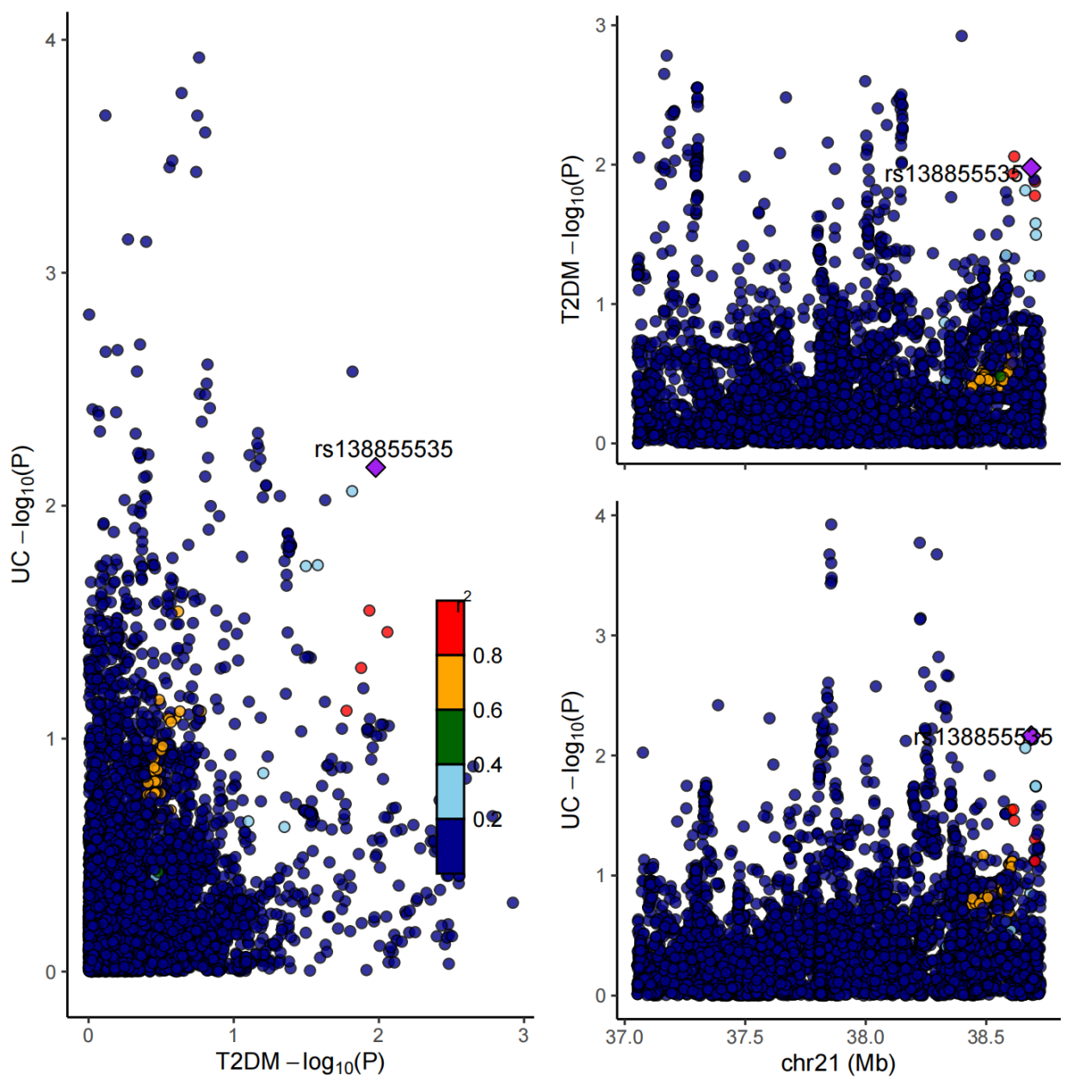


**FIGURE1** Locus2450


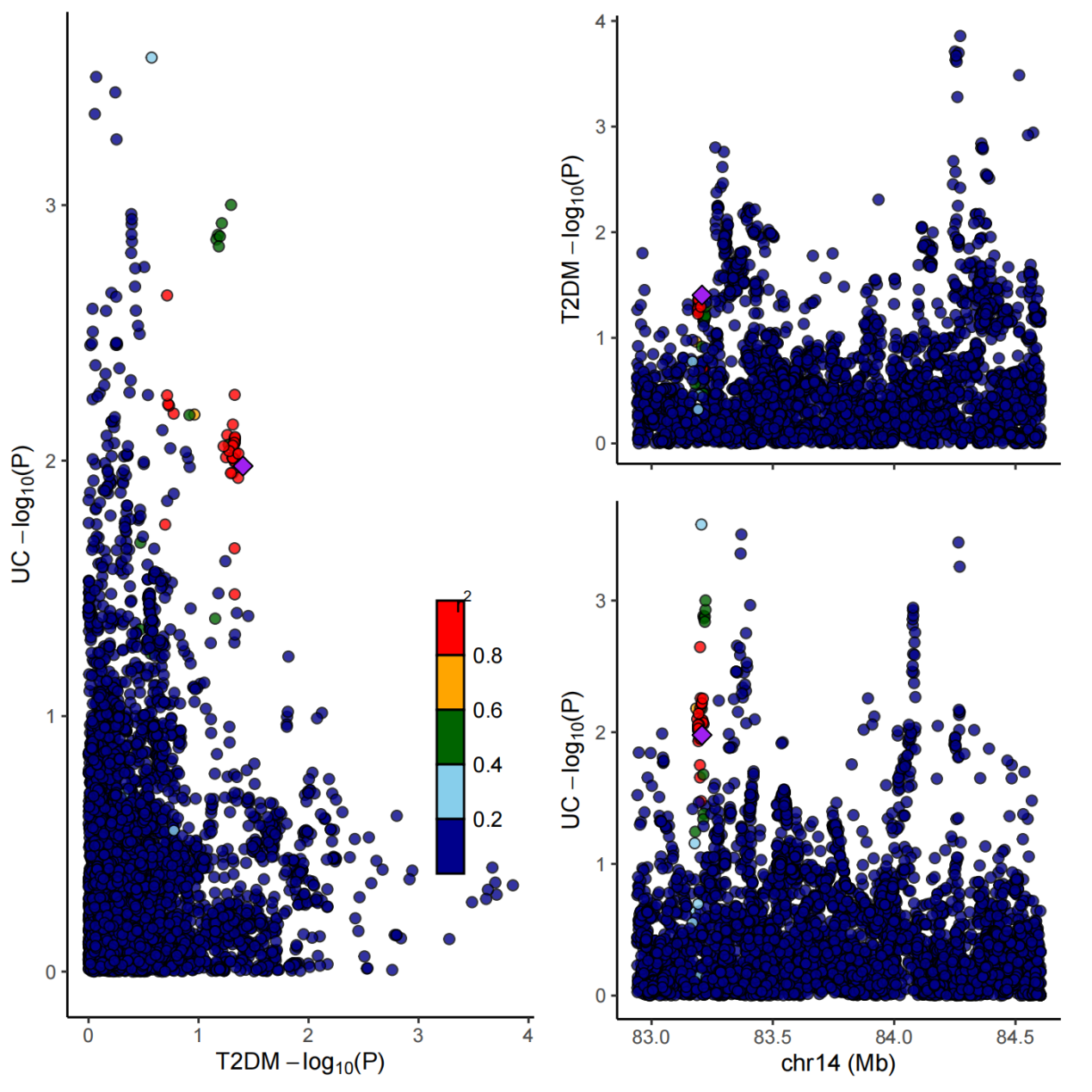


**FIGURE 2** Locus2011


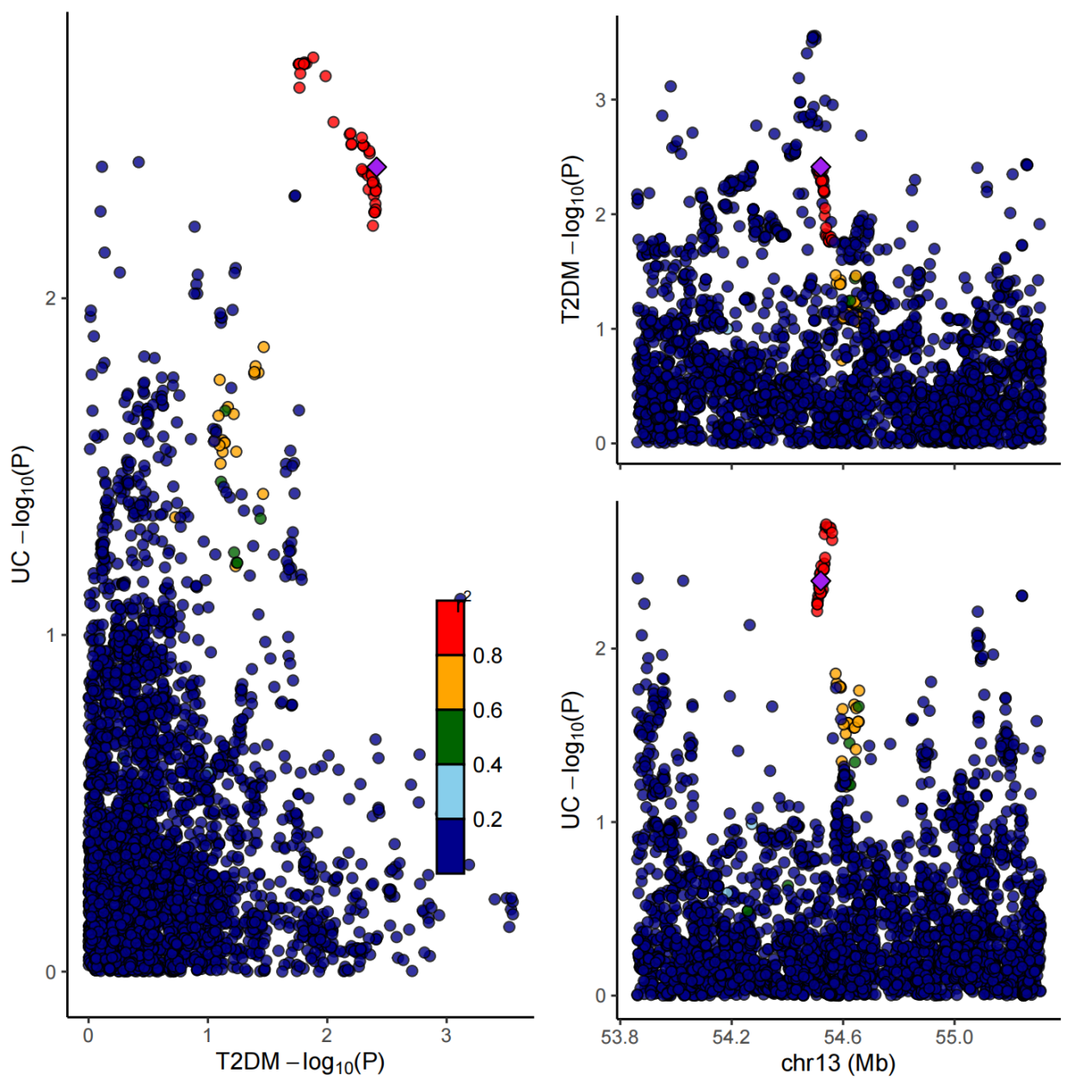


**FIGURE 3** Locus1895


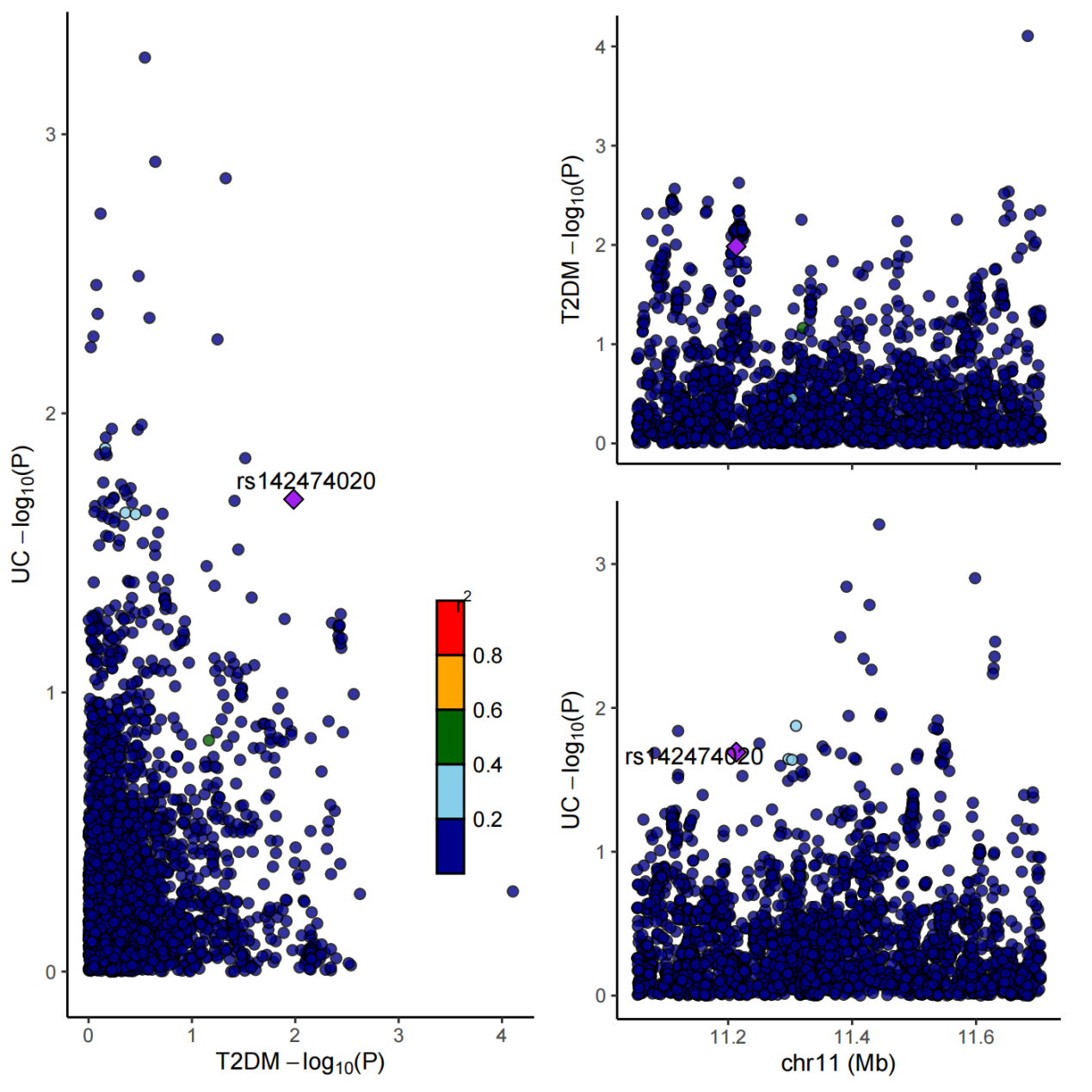


**FIGURE 4** Locus1623


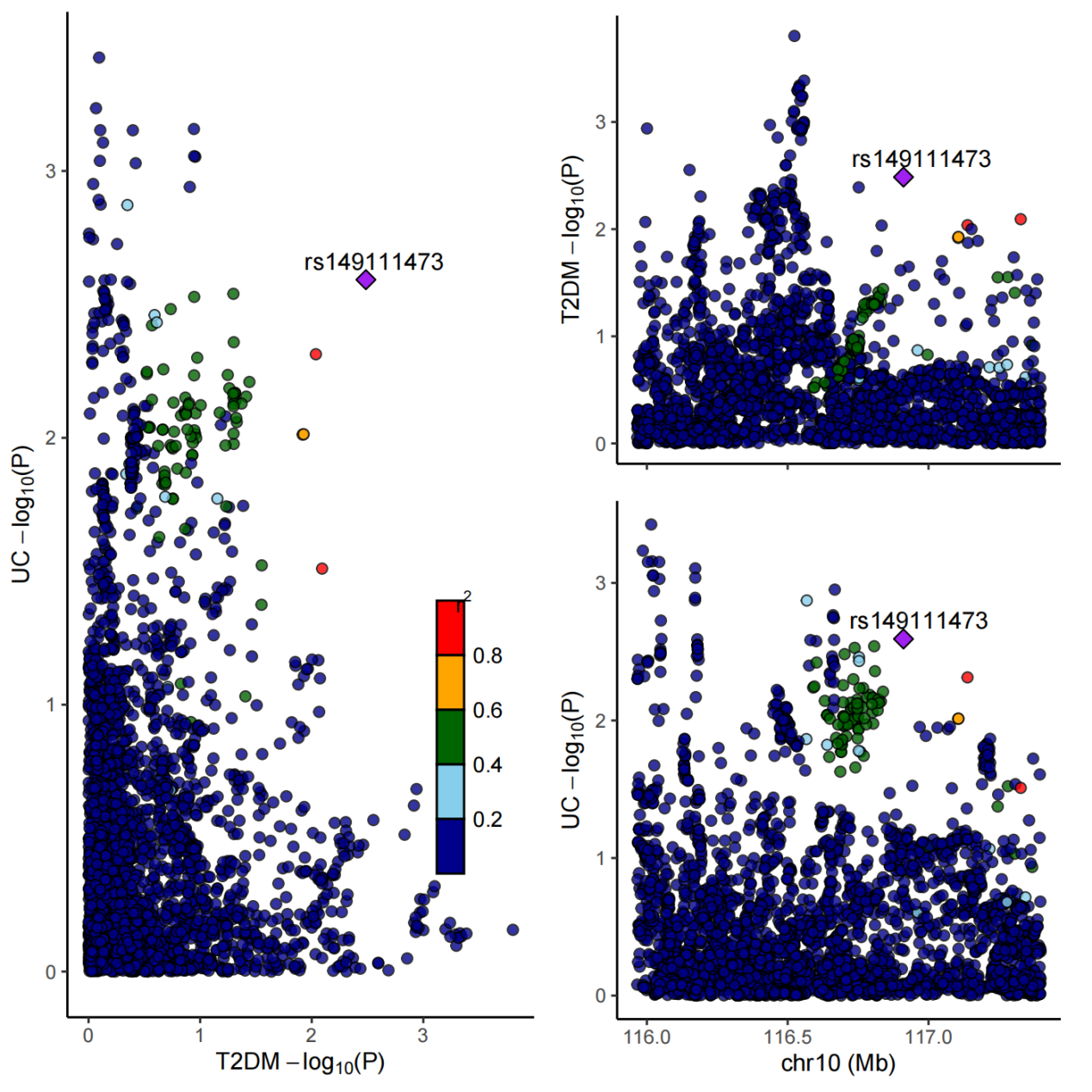


**FIGURE 5** Locus1589


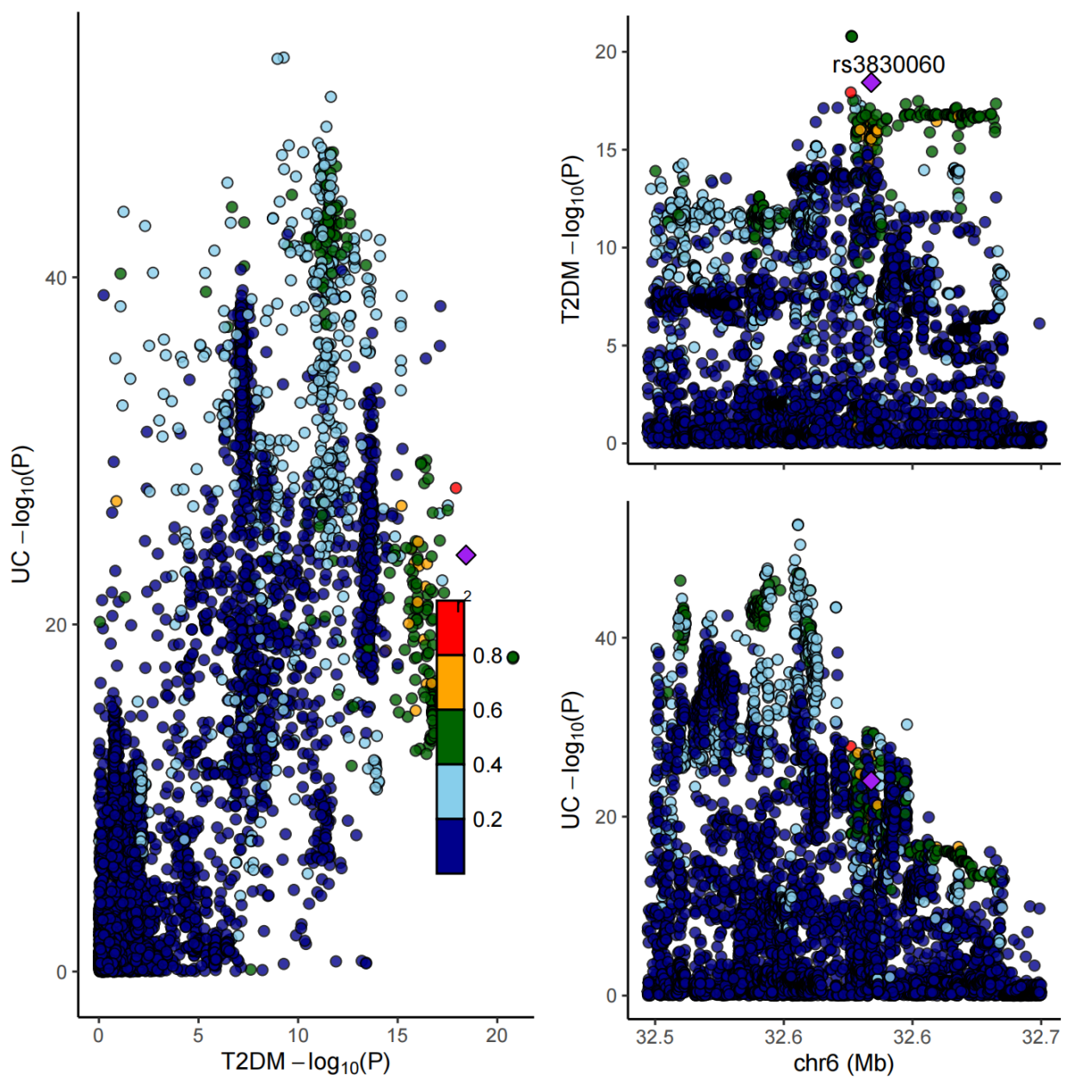


**FIGURE 6** Locus966


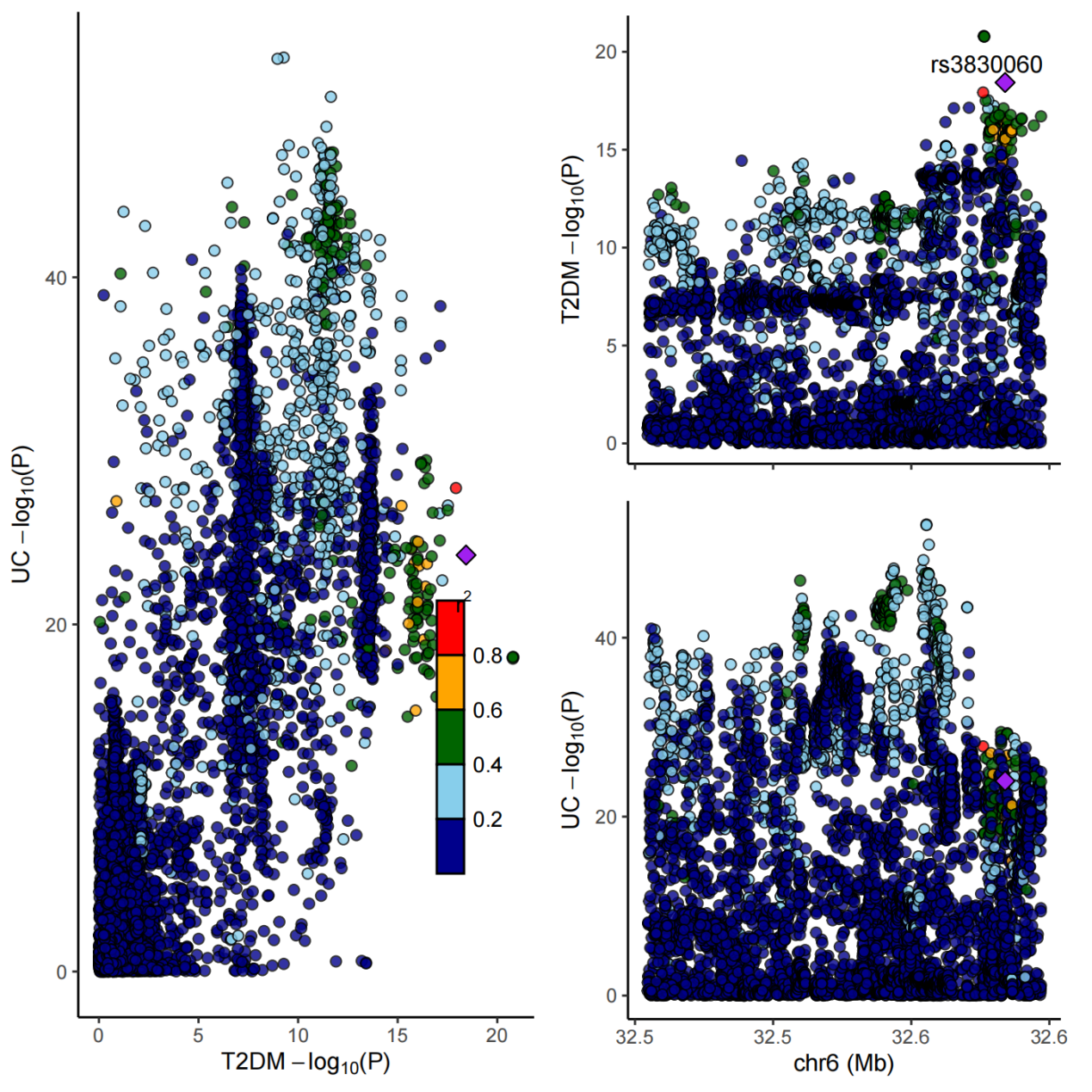


**FIGURE 7** Locus965


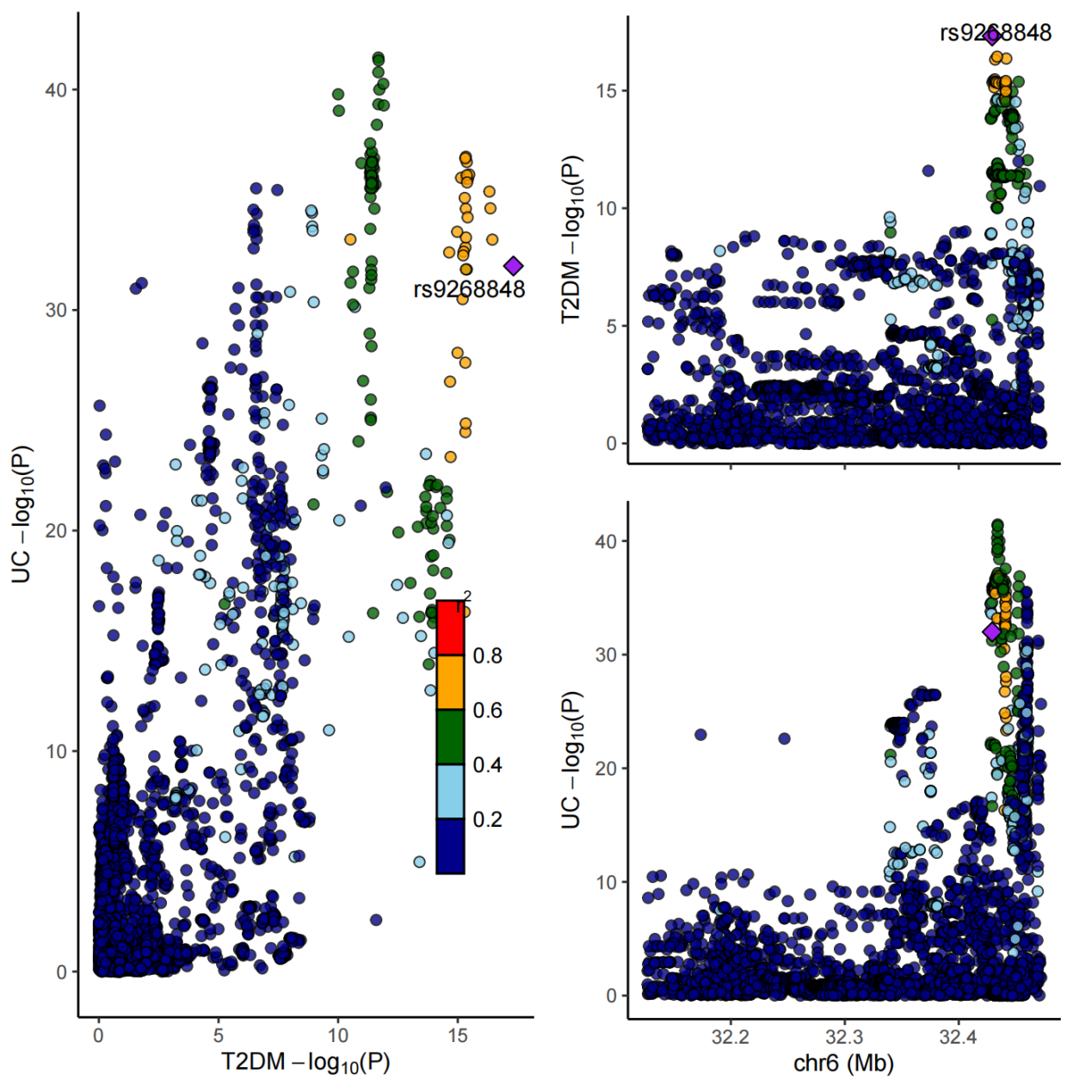


**FIGURE 8** Locus962


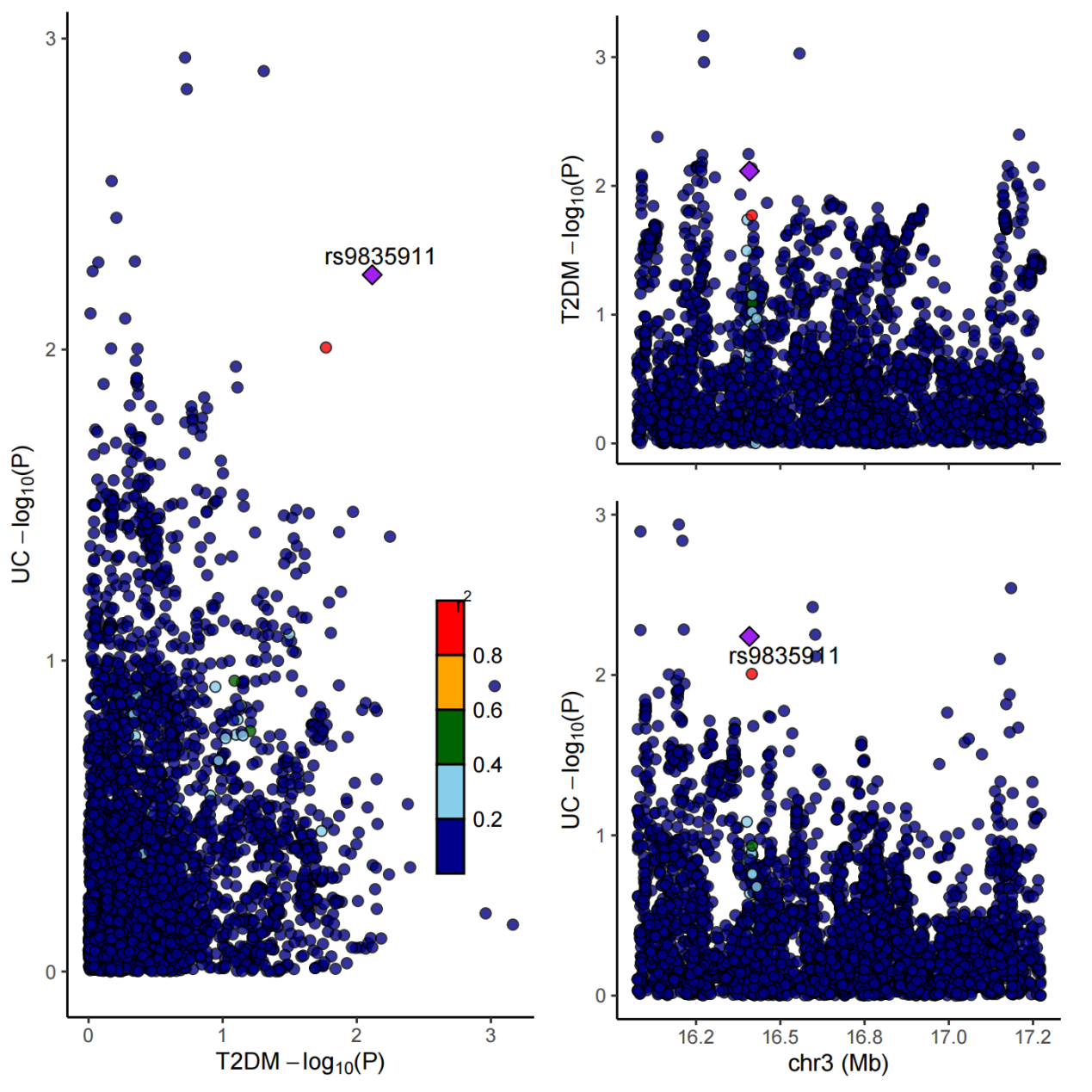


**FIGURE 9** Locus438
